# Using the realized relationship matrix to disentangle confounding factors for the estimation of genetic variance components of complex traits

**DOI:** 10.1186/1297-9686-42-22

**Published:** 2010-06-15

**Authors:** Sang Hong Lee, Michael E Goddard, Peter M Visscher, Julius HJ van der Werf

**Affiliations:** 1 Queensland Statistical Genetics, Queensland Institute of Medical Research, Brisbane, Australia; 2 Biosciences Research Division, Department of Primary Industries, Victoria, Australia; 3 Department of Agriculture and Food Systems, University of Melbourne, Melbourne, Australia; 4 School of Environmental and Rural Science, University of New England, Armidale, Australia

## Abstract

**Background:**

In the analysis of complex traits, genetic effects can be confounded with non-genetic effects, especially when using full-sib families. Dominance and epistatic effects are typically confounded with additive genetic and non-genetic effects. This confounding may cause the estimated genetic variance components to be inaccurate and biased.

**Methods:**

In this study, we constructed genetic covariance structures from whole-genome marker data, and thus used realized relationship matrices to estimate variance components in a heterogenous population of ~ 2200 mice for which four complex traits were investigated. These mice were genotyped for more than 10,000 single nucleotide polymorphisms (SNP) and the variances due to family, cage and genetic effects were estimated by models based on pedigree information only, aggregate SNP information, and model selection for specific SNP effects.

**Results and conclusions:**

We show that the use of genome-wide SNP information can disentangle confounding factors to estimate genetic variances by separating genetic and non-genetic effects. The estimated variance components using realized relationship were more accurate and less biased, compared to those based on pedigree information only. Models that allow the selection of individual SNP in addition to fitting a relationship matrix are more efficient for traits with a significant dominance variance.

## Background

Complex traits are important in evolution, human medicine, forensics and artificial selection programs [1-4]. Most complex traits show a mode of inheritance that may be caused by many functional genes with additive and dominance effects, and possibly epistatic interactions, and environmental effects [5,6].

Traditionally, pedigree information has been used to estimate heritabilities and genetic effects for complex traits [7-10]. In many family studies, non-genetic factors such as familial or shared environmental effects can be confounded with genetic factors [11]. In particular for full-sibs there is confounding between shared environmental effects, additive genetic effects and non-additive genetic effects.

Recently, it has become feasible to generate individual genotype information on large numbers of single nucleotide polymorphisms (SNP) across the whole genome, and genome-wide association studies have been performed in a number of species [12,13]. It is expected that SNP and causal genes will be in linkage disequilibrium (LD), making it possible to genetically dissect variation in complex traits in a more effective way [14]. Indeed, it has been shown that whole-genome dense SNP analyses can provide extra benefits compared to classical approaches based on pedigree information only [15].

In this study, we propose novel strategies that utilize dense SNP data for the genetic dissection of complex traits. First, we estimate a realized relationship matrix based on aggregate SNP information [16-18]. The realized relationship matrix in a classical mixed linear model makes it possible to obtain more accurate and reliable estimates for the narrow sense heritability, compared to traditional pedigree-based analysis [19,20]. Second, we explicitly search for additional additive and dominance effects that may not have been already captured, by using a Bayesian model selection approach. In the process, a stochastic model selection of random SNP effects is carried out nested in a mixed linear model with additive polygenic effects. Additional genetic effects found in this process make it possible to estimate additive genetic and dominance variances with greater precision for some traits which have significant dominance effects. We examine the estimates by using a validation step where unobserved phenotypes in an independent validation set are predicted. We use phenotypic data for four complex traits and genotypic data for ~2200 mice with ~11,000 SNP across the whole genome.

## Methods

### Data

**Figure 1:**
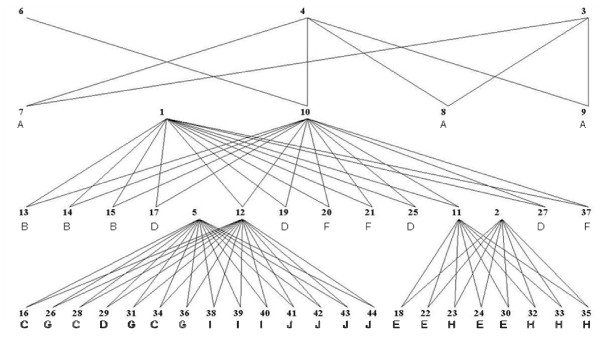
**Family structure for one family among 85 unrelated families**. The members are indexed from 1 to 44; the cage information is under the indexed number if available

Publicly available data including pedigree, genotypic and phenotypic information on heterogeneous stock mice were used [21]; http://gscan.well.ox.ac.uk/. The total number of animals was 2,296 from 85 unrelated families. The available pedigree spanned four generations. In this complex pedigree, there were 172 full-sib families with an average size of ~11 (SD ~8). The mice were reared in a total of 536 cages, and the number of animals per cage ranged from two to seven. This number was considered as a cage density factor for analyses. Figure 1 describes the family structure for one of the 85 unrelated families, which contains 44 members and five nuclear (full-sib) families. Cage information is displayed below each animal when known and indicates a fair degree of confounding between cages and families. Genotypes were available for 12,112 SNP on most animals in the pedigree, and we used the 11,730 SNP located on the autosomal chromosomes. The reason for excluding the sex chromosomes was that modeling them would complicate the analyses without greatly changing the estimates. The phenotypes were already adjusted for environmental fixed effects, e.g. sex, age, year and season [21,22]. However, the effects due to cage, cage density and family were further modeled with and without using information on SNP and additive polygenic effects. Four complex traits were investigated i.e. coat color (CC) (a score from light to dark), weight at 10 weeks (WT), recovery from ear punctuation (REP), and freezing time during cue (FDC). The reasons for choosing these are: CC has a number of major genes with relatively large effects and the environmental variance is small, WT is a typical quantitative trait with the variance probably affected by numerous genes, REP is a quantitative trait with a moderate heritability, and FDC is a quantitative trait with a low heritability.

### Preliminary analysis for each trait

The intra-class correlation of phenotypes for groups having relationship *k *based on pedigree information was estimated (*k *= 1/16, 1/8, 1/4 and 1/2). For example, the intra-class correlation for the group with relationship *k *= 1/2 was that for full-sibs. However, for relationship *k *= 1/16, 1/8, and 1/4, it was difficult to group and classify them because of the complicated pedigree structure. In order to estimate intra-class correlations for the group with relationship *k*, pairs of relationship *k *were used, but in a way that there were no relationships between individuals of different pairs, i.e. relationship = *k *within each pair and relationship = 0 for individuals of different pairs. Because of this restriction, not all pairs of relationship *k *could be used simultaneously. Therefore, we sampled 10,000 independent pairs for each relationship *k *for each trait. The number of pairs for relationship *k*, and the average number of pairs in 10,000 samples are given in Table 1. The variance between these sampled pairs scaled by total variance would be the intra-class correlation [23] for individuals having a relationship *k*. Estimated intra-class correlations were averaged over the 10,000 sampling sets. These correlations are, approximately, the summary statistics that are modeled in the variance component analyses.

**Table 1:** Total number of pairs and average of sampled pairs for relationship *k*

*k*	**FDC**^**a**^		**REP**^**b**^		**WT**^**c**^		**CC**^**d**^	
	total^e^	sample^f^	total	sample	total	sample	total	sample
0	905640		1508985		1686268		1777995	
	(95.2%)		(95.3%)		(95.5%)		(96%)	
1/16	11881	4.3	18429	4.2	19174	4.2	20155	4.2
	(1.3%)	(1.3)	(1.2%)	(1.3)	(1.1%)	(1.3)	(1.1%)	(1.3)
1/8	13598	6.9	21324	6.8	23204	6.7	24215	6.6
	(1.4%)	(2.3)	(1.5%)	(2.2)	(1.3%)	(2.2)	(1.3%)	(2.2)
1/4	6530	6.8	11823	7.6	13303	7.8	13977	7.7
	(0.7%)	(2.3)	(0.8%)	(2.8)	(0.8%)	(2.8)	(0.8%)	(2.9)
1/2	9063	42.5	13288	43.8	14556	44.1	15273	44.4
	(0.9%)	(2.4)	(0.8%)	(2.3)	(0.9%)	(2.2)	(0.8%)	(2.2)
	951510		1583310		1766260		1861485	
	(100%)		(100%)		(100%)		(100%)	

In estimating intra-class correlations for relationships *k*, the total number of pairs (%), and the average number of sampled pairs (standard deviation) in 10,000 samples for each *k *for each trait

^a^Freezing during cue; ^b^Recovery from ear punctuation; ^c^Weight at 10 weeks; ^d^Coat color ^e^Total number of pairs for each relationship for each trait; ^f^Average number of pairs in 10,000 samples

### Mixed linear model implementing a numerator relationship matrix based on pedigree information

A mixed linear model analysis was used to estimate random polygenic, cage and family effects, and the fixed effect of cage density. The model can be expressed as,

where **y **is a vector of *N*_*r *_phenotypic observations, **β **is a vector of fixed effects including the overall mean and the cage density as covariates, **f **is a vector of *N*_*f *_random environmental family effects, **c **is a vector of *N*_*c *_random environmental cage effects, **u **is a vector of *N *random additive polygenic effects for all animals derived from pedigree information (*N *= 2296), and **e **is a vector of *N*_*r *_residuals. It is assumed that **f, c **and **u **are normally distributed with a mean of 0 and a variance of ,  and , respectively. **X**, **W**, **U **and **Z **are incidence matrices for the effects. The variance covariance matrix (**V**) of phenotypic observations for the model can be written as,

where **A **is the numerator relationship matrix based on pedigree information only, and **I **is an identity matrix. In order to see if estimates for genetic and environmental family effects are dependent, a simple comparison is carried out for model 1, by omitting subsequently the term **u **(model 1**-u**) or *f *(model 1**-f**). Variance components and effects are estimated by a residual maximum likelihood (REML) method [24,25]. The ratio of each variance component over the total phenotypic variance was calculated.

### Mixed linear model implementing a realized relationship matrix based on genome wide SNP information

When SNP information is available, the realized relationship matrix (**G**) can be estimated and implemented in the model [16-18]. To estimate **G**, we used the method introduced by Oliehoek et al. (2006) since it is robust and best-performed among tested methods in their study. The details to estimate **G **are in Appendix A. The model can be written as,

where **g **is a vector of *N *random genome-wide effects for all animals. It was assumed that **g **is normally distributed with mean 0 and variance . The variance covariance matrix of phenotypic observations for this model is,

Variance components and effects were again estimated by REML [24,25].

### Bayesian approach to model specific SNP effects

Effects of specific quantitative trait loci (QTL) may not be fully captured by model 2, and a Bayesian approach can be used to explicitly search for sets of SNPs that explain additional genetic variance. In the first instance, we model only additive effects of QTL. The model can be written as,

where *n*_*q *_is the number of SNP associated with the QTL, ∝_*i *_is the random additive effects of the i^th ^SNP which is normally distributed with mean 0 and variance , **Λ **_*i *_is a column vector having coefficients 0, 1 or 2 representing indicator variables of the genotype for each animal at the i^th ^SNP. The variance covariance matrix of phenotypic observations is,

In addition to additive SNP effects, dominant SNP effects are modeled for SNP having three genotypes and its heterozygosity > 10%. The model can be written as,

where σ_*i *_is the random dominance effects of the i^th ^SNP assuming a normal distribution with mean 0 and variance , and **Δ**_*i *_is a column vector having coefficients equal to 1 for a heterozygous genotype and 0 for a homozygous genotype at the i^th ^SNP. The variance covariance matrix of phenotypic observations is,

The polygenic heritability based on **G**, and the ratio of variance due to family, cage and additive and dominance SNP effects over the total phenotypic variance were estimated using a reversible jump Markov chain Monte Carlo (RJMCMC) and REML.

In the estimation of variance components, solving mixed model equation (MME) was a heavy computing task because of very dense **G**. Therefore, solving dense MME and obtaining REML estimates in every MCMC round was almost impossible in models 3 and 4. Because of this obstacle, we used a computationally tractable strategy to estimate variance components. Initially, variance components were estimated using REML from model 2 (, , and ). In an RJMCMC process (Appendix B), the number of SNP associated with QTL, their positions and effects were sampled, conditional on the estimated variance components of , , and . The SNP effects were treated as fixed effects such that it was not required to update the variance covariance matrix (**V**) nor invert **V **for each set of sampled QTL effects, which made it possible to carry out a large number of RJMCMC rounds. Variance components for family, cage, polygenic and additive and dominance SNP effects were estimated every 1000 rounds using REML, and the estimated variance components were stored to obtain the posterior mean of the estimates. We used a total of 100,000 rounds of MCMC after 10,000 burn-in periods. Although the variance components were updated and stored only 100 times, the estimates reached convergence quickly probably because of a large number of iterations for the main process.

In order to efficiently search for sets of significant SNP, we preliminarily pruned SNP, and excluded closely linked SNP having r_2 _> 0.95 in sliding 50 SNP windows using PLINK [26]. After pruning, 4194 SNP remained and were used for the Bayesian analysis.

### Validation of estimates (predicting unobserved phenotypes)

We predicted phenotypes of individuals (**ŷ **) with models 1 to 4. In the Bayesian approach (models 3 and 4), averages of **ŷ ** over all RJMCMC rounds were used as predicted phenotypes. In order to quantify how well each model can disentangle genetic effects from environmental effects, we used two strategies to produce estimation and validation sets. First, we randomly selected approximately half of the individuals within each full-sib family, which divided the whole data into two subsets. One set was used as an estimation set, and the other set was used as a validation set. Since some individuals in the estimation and validation sets belonged to the same full-sib family, prediction was carried out within full-sib families. Second, approximately half of the full-sib families were randomly selected within each of the 85 unrelated families. This also divided the whole data into two subsets. In this case, no individual in the estimation and validation sets shared the same full-sib family although they would be related. Therefore, prediction was performed across full-sib families.

In ten replicates, the phenotypes for a validation set (~50% of the population) were predicted from the estimation based on the phenotypes and genotypes for the rest of the population in the estimation set. For each comparison, we correlated the predicted value of an animal in the validation set with its phenotype (which was not used in the estimation phase). We term the correlation between predicted phenotypes and actual phenotypes as the accuracy of prediction.

## Results

### Intra-class correlation

**Figure 2:**
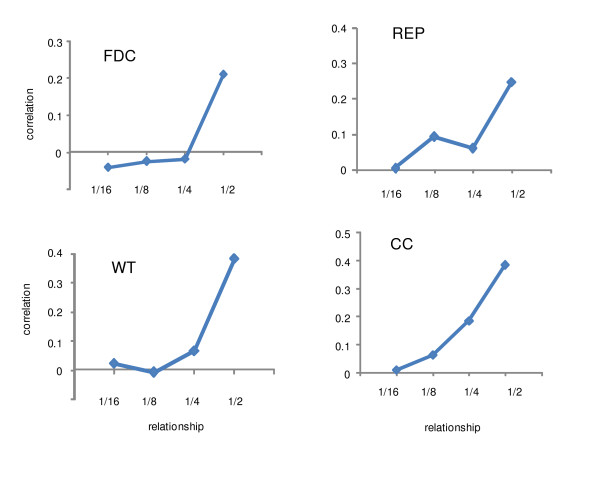
**Intra-class phenotypic correlation**. Intra-class phenotypic correlation plotted against relationship based on pedigree information. FDC - Freezing during cue; REP - Recovery from ear punctuation; WT - Weight at 10 weeks; CC - Coat color

Figure 2 shows phenotypic correlations as a function of additive relationship for each trait. For all traits, the correlation among full-sibs (k = 1/2) was relatively much higher than for other types of relationship. For CC, the correlation increased exponentially. For REP, the correlations for *k *= 1/16, 1/8 and 1/4 were relatively low and there was little increase until a highly increased correlation for *k *= 1/2. For FDC, the correlations for *k *= 1/16, 1/8 and 1/4 were close to zero with again a much higher value for *k *= 1/2. For WT, the pattern was similar; the correlations for 1/16, 1/8 and 1/4 were low, and not much different from each other, but increased dramatically with *k *= 1/2. The relative high correlations for *k *= 1/2 were probably due to the fact that members within this group (i.e. full-sib) had common dominance and environmental family effects in addition to common additive genetic effects.

### Estimating variance components

Estimated variance components proportional to the total phenotypic variance and model log-likelihood are compared in Tables 2, 3, 4 and 5. The results for the trait FDC are shown in Table 2. The model without family effects gave a log-likelihood value of 1619.24 which was significantly lower than that from the full model 1. A model without polygenic effects gave the same log-likelihood as the full model (1621.3), indicating that no genetic effects are captured by the pedigree information. Indeed, genetic variance was estimated as zero in the full model 1. This was not the case in model 2 which implemented the realized relationship matrix based on aggregate SNP information. In model 2, the variance due to additive genetic effects was increased to 25%, and the variance due to family effects was decreased to 7% of the total phenotypic variance. The model log-likelihood increases to 1633.91 which was much higher than that from model 1. This showed that the realized relationship matrix based on SNP information could disentangle the genetic effects which were confounded with environmental family effects in the pedigree-based analysis. When using model 3 to search for specific additive SNP effects, the additive genetic variance increased slightly to 30% of total phenotypic variance, e.g. 18% due to polygenic and 12% due to specific SNPs. The variances for family and cage effects did not change much compared to model 2. The averaged log-likelihood was 1650.56, and the averaged number of QTL fitted in the models was 3.55 in the RJMCMC process. When using model 4 to search for specific additive and dominant SNP effects, a relatively large variance due to dominance effects was estimated (27% of total phenotypic variance). Model 4 showed the highest value for the average log-likelihood, and the average number of additive and dominance QTL fitted was 10.2. The averaged Akaike information criterion (AIC) for model 4 was dramatically lower than that for model 3, implying that model 4 was not better than model 3.

The results for the trait REP are shown in Table 3. A model without either polygenic effects or environmental family effects gave a lower log-likelihood than the full model 1. This indicated that both polygenic and family effects should be fitted in the model. In the full model 1, the variance of family, cage and polygenic effects as percentage of total phenotypic variance was 10%, 11% and 25%, respectively. When using model 2, the additive genetic variance increased to 50% of total phenotypic variance, while family and cage variance was reduced to 6% and 8% of total phenotypic variance, respectively. The log-likelihood with model 2 was substantially higher than that with model 1 (1670.71). This indicated that the model implementing the realized relationship matrix based on aggregate SNP information explained variation in phenotypes better than the model implementing the numerator relationship matrix based on pedigree information (this is also empirically proven in the next section). When using model 3, the estimated variance due to additive genetic effects increased slightly to 54% of total phenotypic variance. Variances for family and cage effects did not change much compared to those of model 2. The average log-likelihood was 1717.3, and the average number of QTL was 5.3 in the RJMCMC process. When using model 4, the estimated dominance variance was 15% of total phenotypic variance. The average log-likelihood was 1730.33 and the average number of additive and dominance QTL was 14.72. The average AIC for model 4 was not much improved, compared to that for model 2 (Table 3).

**Table 2:** Estimated parameters for FDC

	Model 1	**Model 1-u**^a^	**Model 1-f**^b^	Model 2	Model 3	Model 4
*f*^2^	0.14	0.14	N/A	0.07	0.06	0.03
	(0.03)	(0.03)		(0.03)	(0.02)	(0.02)
*c*^2^	0.02	0.02	0.03	0.02	0.02	0.01
	(0.03)	(0.03)	(0.03)	(0.02)	(0.02)	(0.01)
*u*^2^	0.00	N/A	0.29	N/A	N/A	N/A
	(0)		(0.06)			
*g^2 ^*	N/A	N/A	N/A	0.25	0.18	0.10
				(0.06)	(0.06)	(0.05)
*α*^2^	N/A	N/A	N/A	N/A	0.12	0.21
					(0.13)	(0.22)
*δ*^2^	N/A	N/A	N/A	N/A	N/A	0.27
						(0.23)
Log L	1621.30	1621.30	1619.24	1633.91	1650.56^c^	1695.96^d^
					(3.55)	(10.17)
Parameters	**f, c, u **	**f, c **	**c, u **	**f, c, g **	**f, c, g, α **	**f, c, g, α, δ**
AIC^e^	-3236.60	-3238.60	-3234.48	-3261.82	-3288.02	-3365.58

Proportion of total phenotypic variance due to family (**f**), cage (**c**), and polygenic effects based on pedigree (**u**), realized relationships (**g**), and specific additive and dominance SNP effects (**α **and **δ**) when using model 1, 2, 3 and 4 for FDC

^a^Model 1 without the term **u**, ^b^Model 1 without the term **f**, ^c^The averaged log-likelihood during MCMC process (the averaged number of parameters due to additive SNP in the model), ^d^The averaged likelihood during MCMC process (the averaged number of parameters due to additive and dominance SNP in the model), ^e^AIC = 2 * number of parameters - 2 * log likelihood

**Table 3:** Estimated parameters for REP

	Model 1	**Model 1-u**^a^	**Model 1-f**^b^	Model 2	Model 3	Model 4
*f*^2^	0.1	0.22	N/A	0.06	0.05	0.04
	(0.07)	(0.03)		(0.02)	(0.02)	(0.02)
*c*^2^	0.11	0.11	0.12	0.08	0.08	0.06
	(0.02)	(0.02)	(0.02)	(0.02)	(0.02)	(0.02)
*u*^2^	0.25	N/A	0.46	N/A	N/A	N/A
	(0.15)		(0.07)			
*g*^2^	N/A	N/A	N/A	0.50	0.36	0.29
				(0.04)	(0.08)	(0.08)
*α*^2^	N/A	N/A	N/A	N/A	0.18	0.20
					(0.16)	(0.17)
*δ*^2^	N/A	N/A	N/A	N/A	N/A	0.15
						(0.14)
Log L	1604.08	1602.37	1602.88	1670.71	1717.3^c^	1730.33^d^
					(5.26)	(14.72)
Parameters	**f, c, u **	**f, c **	**c, u **	**f, c, g **	**f, c, g, α **	**f, c, g, α, δ **
AIC^e^	-3202.16	-3200.74	-3201.76	-3334.20	-3418.08	-3425.22

Proportion of total phenotypic variance due to family (**f**), cage (**c**), and polygenic effects based on pedigree (**u**), realized relationships (**g**), and specific additive and dominance SNP effects (**α **and **δ**) when using model 1, 2, 3 and 4 for REP

^c^The averaged log-likelihood during MCMC process (the averaged number of parameters due to additive SNP in the model)

^d^The averaged log-likelihood during MCMC process (the averaged number of parameters due to additive and dominance SNP in the model)

Table 4 shows the results for the trait WT. On the one hand, the model without polygenic effects gave a log-likelihood of 3382.73 which was significantly lower than that from the full model 1 (3389). On the other hand, the family effects were shown to be negligible in phenotypic variation, i.e. a reduced model excluding family effects gave the same likelihood as the full model. In the full model 1, the family, cage and polygenic variances were estimated as 0%, 17% and 64% of total phenotypic variance, respectively. However, model 2 gave very different estimates, i.e. 14%, 16% and 38% for family, cage and polygenic variances, respectively. The log-likelihood for model 2 was much higher than that for model 1. When using model 3, the family and cage variances decreased slightly to 12% and 14% while the additive genetic variance increased to 48%, e.g. 27% due to polygenic and 21% due to specific SNPs. The values for the average log-likelihood and AIC were improved although they were not substantially higher than those for model 2. In model 4, the family and cage variances decreased to 5% and 6%. The additive genetic variance was 44% which was not very different to that of model 3, and the dominance variance was estimated as 35%. The average log-likelihood and AIC were moderately improved.

**Table 4:** Estimated parameters for WT

	Model 1	**Model 1-u**^a^	**Model 1-f**^b^	Model 2	Model 3	Model 4
*f*^2^	0.00	0.32	N/A	0.14	0.12	0.05
	(0.00)	(0.04)		(0.03)	(0.03)	(0.03)
*c*^2^	0.17	0.17	0.17	0.16	0.14	0.06
	(0.02)	(0.02)	(0.02)	(0.02)	(0.03)	(0.03)
*u*^2^	0.64	N/A	0.65	N/A	N/A	N/A
	(0.08)		(0.08)			
*g*^2^	N/A	N/A	N/A	0.38	0.27	0.10
				(0.04)	(0.06)	(0.06)
*α*^2^	N/A	N/A	N/A	N/A	0.21	0.34
					(0.16)	(0.27)
*δ*^2^	N/A	N/A	N/A	N/A	N/A	0.35
						(0.31)
Log L	3389.00	3382.73	3389.00	3438.03	3464.47^c^	3499.46^d^
					(5.59)	(19.46)
Parameters	**f, c, u **	**f, c **	**c, u **	**f, c, g **	**f, c, g, α **	**f, c, g, α, δ **
AIC^e^	-6772.00	-6761.46	-6774.00	-6870.06	-6911.76	-6954.00

Proportion of total phenotypic variance due to family (**f**), cage (**c**), and polygenic effects based on pedigree (**u**), realized relationships (**g**), and specific additive and dominance SNP effects (**α **and **δ**) when using model 1, 2, 3 and 4 for WT

^c^The averaged log-likelihood during MCMC process (the averaged number of parameters due to additive SNP in the model)

^d^The average log-likelihood during MCMC process (the averaged number of parameters due to additive and dominance SNP in the model)

The results for the trait CC are shown in Table 5. A model without polygenic effects based on pedigree information gave a significantly lower log-likelihood compared to the full model 1 but omitting family effects gave only a small change. When using model 2, there were only slight changes in the variance components, e.g. the family variance increased to 7% and the polygenic variance decreased slightly to 71% of total phenotypic variance. However, the model log-likelihood was considerably higher than that from the model 1. When using model 3, the estimated variances were similar to those of model 2 although most of the additive genetic variance was captured by specific SNP. In model 4, nearly all the variance was captured by additive and dominant QTL effects and the averaged log-likelihood as well as AIC were far better than in any of the other models.

**Table 5:** Estimated parameters for CC

	Model 1	**Model 1-u**^**a**^	**Model 1-f**^**b**^	Model 2	Model 3	Model 4
*f*^2^	0.00	0.36	N/A	0.07	0.08	0.01
	(0.00)	(0.03)		(0.02)	(0.04)	(0.01)
*c*^2^	0.01	0.02	0.01	0.00	0.00	0.00
	(0.01)	(0.02)	(0.01)	(0.01)	(0.01)	(0.00)
*u*^2^	0.73	N/A	0.73	N/A	N/A	N/A
	(0.07)		(0.07)			
*g*^2^	N/A	N/A	N/A	0.71	0.05	0.01
				(0.03)	(0.03)	(0.01)
*a*^2^	N/A	N/A	N/A	N/A	0.66	0.47
					(0.17)	(0.37)
*δ*^2^	N/A	N/A	N/A	N/A	N/A	0.49
						(0.37)
Log L	-2373.14	-2381.68	-2373.14	-2190.39	-1954.99^c^	-974.46^d^
					(7.88)	(29.48)
Parameters	**f, c, u**	**f, c**	**c, u**	**f, c, g**	**f, c, g, α**	**f, c, g, α, δ**
AIC^e^	4752.28	4767.36	4750.26	4387.84	3931.74	2013.88

Proportion of total phenotypic variance due to family (**f**), cage (**c**), and polygenic effects based on pedigree (**u**), realized relationships (**g**), and specific additive and dominance SNP effects (**α ** and **δ **) when using model 1, 2, 3 and 4 for CC

^c^The averaged log-likelihood during MCMC process (the averaged number of parameters due to additive SNP in the model)

^d^The average log-likelihood during MCMC process (the averaged number of parameters due to additive and dominance SNP in the model)

### Correlation between estimated variance components

Table 6 shows sampling correlations between estimated variance components as derived from the average information matrix, i.e. the variance covariance matrix of estimated variance components. Correlations between **f **and **u **were very high and negative for REP, WT and CC, ranging from -0.85 to -0.94. Correlations between **c **and **u **were moderate and negative for FDC (-0.41). This showed that the additive genetic effects derived from pedigree information were highly confounded with the environmental family or cage effects. However, correlations between **f **and **g **were low for all the traits (-0.1 ~ -0.23), and those between **c **and **g **were negligible, indicating that realized relationships based on aggregate SNP information could disentangle genetic effects from environmental effects. For all the traits, the sampling correlations between estimated variances due to genetic and non-genetic effects were close to zero when using models 3 and 4.

**Table 6:** Sampling correlation between estimated variance components

	Model	(f, c)	(f, u)	(c, u)	(f, g)	(c, g)	(f, snp)	(c, snp)	(g, snp)
FDC	1	-0.28	-0.02	-0.41	N/A	N/A	N/A	N/A	N/A
	2	-0.28	N/A	N/A	-0.22	-0.01	N/A	N/A	N/A
	3	-0.28	N/A	N/A	-0.23	-0.01	-0.01	0	0
	4	-0.29	N/A	N/A	-0.23	-0.02	0	0	0
REP	1	-0.09	-0.85	-0.01	N/A	N/A	N/A	N/A	N/A
	2	-0.24	N/A	N/A	-0.17	0.01	N/A	N/A	N/A
	3	-0.25	N/A	N/A	-0.18	0.01	0	0	0
	4	-0.26	N/A	N/A	-0.18	0.01	-0.01	0.01	0.01
WT	1	0.11	-0.94	-0.2	N/A	N/A	N/A	N/A	N/A
	2	-0.22	N/A	N/A	-0.1	0.01	N/A	N/A	N/A
	3	-0.23	N/A	N/A	-0.11	0.02	0	0	0
	4	-0.23	N/A	N/A	-0.11	0.02	-0.01	0.01	0
CC	1	-0.09	-0.91	-0.09	N/A	N/A	N/A	N/A	N/A
	2	-0.11	N/A	N/A	-0.11	-0.02	N/A	N/A	N/A
	3	-0.11	N/A	N/A	-0.16	-0.05	0	0	0
	4	-0.11	N/A	N/A	-0.19	-0.09	0	0	0

Correlation between estimated variance components for family (**f**), cage (**c**), polygenic effects based on pedigree (**u**) and realized relationships (**g**), and specific SNP effects (*snp*) when using model 1, 2, 3 and 4.

### Validating estimates and prediction of unobserved phenotypes

Accuracies of the prediction of unobserved phenotypes for the various models are shown in Table 7. Prediction was carried out for individuals within full-sib families or across full-sib families. In general, the accuracy was much lower for model 1 than for model 2. For all the traits, the accuracies for model 3 were slightly higher than those for model 2 although the differences in accuracy between models 2 and 3 were not significant. For FDC and CC the accuracies for model 4 were far better than those for model 3 where there was a considerable difference in AIC between models 3 and 4. However, for REP and WT there was no significant difference between the accuracies for models 3 and 4 and AIC values for the models were also not substantially different to each other. Accuracies were highest for CC, which has the largest heritability, and smallest for FDC which has also the lowest heritability.

**Table 7:** Accuracy (acc) and regression (reg) in the prediction of phenotypes

Trait	Model 1		Model 2		Model 3		Model 4	
	acc	reg	acc	reg	acc	reg	acc	reg
	prediction within full-sib families							
FDC	0.21	1.03	0.26	0.94	0.28	0.87	0.35	0.87
	(0.04)	(0.4)	(0.04)	(0.16)	(0.05)	(0.19)	(0.03)	(0.08)
REP	0.44	1.01	0.51	0.99	0.52	0.95	0.52	0.93
	(0.02)	(0.10)	(0.02)	(0.07)	(0.02)	(0.06)	(0.02)	(0.07)
WT	0.54	1	0.57	0.97	0.57	0.95	0.56	0.93
	(0.03)	(0.13)	(0.03)	(0.06)	(0.03)	(0.09)	(0.04)	(0.1)
CC	0.54	0.97	0.65	0.97	0.69	0.93	0.87	0.99
	(0.02)	(0.06)	(0.01)	(0.05)	(0.01)	(0.05)	(0.03)	(0.02)
prediction across full-sib families								
FDC	-0.02	-0.53	0.16	0.89	0.19	0.84	0.29	0.85
	(0.04)	(1.94)	(0.04)	(0.23)	(0.07)	(0.27)	(0.05)	(0.08)
REP	0.12	0.83	0.31	0.81	0.36	0.8	0.35	0.75
	(0.05)	(0.4)	(0.04)	(0.15)	(0.05)	(0.14)	(0.04)	(0.13)
WT	0.18	0.97	0.3	0.97	0.3	0.87	0.28	0.76
	(0.04)	(0.28)	(0.03)	(0.13)	(0.04)	(0.2)	(0.06)	(0.23)
CC	0.17	0.85	0.46	0.9	0.53	0.8	0.78	0.95
	(0.04)	(0.2)	(0.07)	(0.17)	(0.09)	(0.17)	(0.09)	(0.13)

The average of correlations of actual and predicted phenotypes (standard deviations), and regression of the true phenotypes on predicted phenotypes (standard deviations) over 10 replicates when using model 1, 2, 3 and 4 for the traits

The accuracies for predicting individuals within full-sib families were higher than those for predicting across full-sib families, which was expected since family information could not be used across the full-sib families. Interestingly, the difference between the accuracies for models 1 and 2 was larger when predicting phenotypes across full-sib families, compared to that when predicting phenotypes within full-sib families. The reduction in accuracy due to lack of family information was larger when using model 1 than when using model 2. This showed that the performance of model 2 was apparently less dependent on environmental family effects.

Deviation from unity of the regression coefficient of true phenotypes on predicted phenotypes is an indication of bias in the estimation compared to the true value. The averaged values of regression coefficients were close to 1 when predicting phenotypes within full-sib families. However, when predicting phenotypes across full-sib families, the values were clearly biased probably because of lack of family information across the full-sib families. In general, models 3 and 4 would give more biased estimates, compared to models 1 or 2 although the difference was small.

## Discussion

We have shown that a mixed linear model implementing a realized relationship matrix based on aggregate SNP information can efficiently disentangle genetic effects from environmental family and cage effects when the number of causal genes is large and their effects are additive, e.g. REP and WT in this study. When dealing with a trait having a limited number of causal genes with possibly dominance effects, e.g. FDC and CC in this study, a model with a finite number of individual loci can be used to help to disentangle efficiently genetic effects from non-genetic effects. Moreover, the latter model can separate additive and non-additive genetic effects and capture more of the total genetic variance. Therefore, the estimated variance components and resulting solutions from the models based on SNP information are more reliable and accurate, compared to those based on pedigree information only, and they allow a better dissection of the various genetic and non-genetic components of variation.

For REP and WT there was no improvement in accuracies for models 3 or 4, compared to those for model 2, which may be due to the fact that the true model for the traits is probably an infinitesimal model like model 2, i.e. a large number of causal genes, each with a small effect. Another possible reason might be that we used a slightly unrealistic prior for the number of QTL in the RJMCMC process. We used a Poisson distribution with a mean of 1 as the prior distribution for the number of QTL (Appendix B). It has been reported previously that the method is robust to different priors for the number of QTL [15,27,28]. Higher values gave more QTL sampled into the model, but the effect on prediction accuracy was small [15].

Since we analysed a single data set we cannot be sure about all the causal factors and how they are (partially) confounded. However, we have shown that the model likelihood increased (Tables 2 to 5), the sampling correlation between estimated effects for the factors decreased (Table 6), and the accuracy of predicting genetic effects in validation sets increased (Table 7) when using the models based on whole-genome SNP data. These observations strongly suggest that confounding effects between genetic and non-genetic effects are better disentangled when using whole-genome SNP data, compared to traditional approaches based on pedigree information only.

In our study, we have estimated a variance covariance matrix of the variance components using average information from Fisher's scoring and the Hessian matrix [25]. A full Bayesian approach [29-31] may be able to assess the confounding between family, cage and polygenic effects by estimating the posterior correlations between variance components, e.g. BUGS [32]. Our approach differs from a full Bayesian method as we used a (residual) maximum likelihood within the MCMC process to take advantage of a quick convergence and to decrease reducibility problems. Moreover, the realized relationship matrix was simultaneously fitted with specific SNP effects so that larger SNP effects, with or without dominance effects, could be captured and estimated adjusted for polygenic effects. In real practical situations where genetic and environmental effects are often confounded, the proposed approach may be worthwhile to implement and help dissect genetic variation of complex traits.

**Figure 3:**
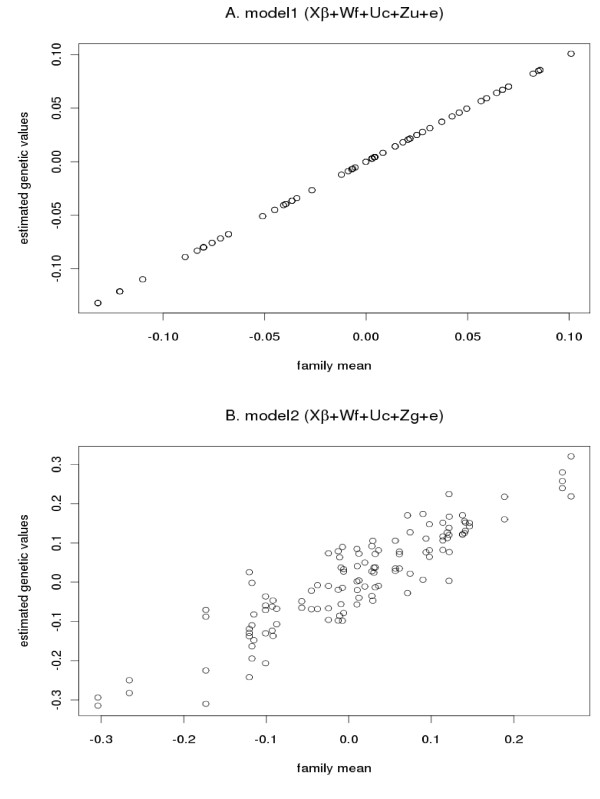
**Variation of estimated genetic values within families**. Estimated genetic values plotted against family mean for model 1 (A) and model 2 (B)

The better performance of the realized relationship matrix based on SNP information, compared to the numerator relationship matrix based on pedigree, is probably due to the fact that SNP-based analysis can better predict some of the variation within a family [16]. In Figure 3, a validation set for REP was used as an example to show variation in estimated genetic values within families. As shown in Figure 3, individual genetic values estimated from model 1 based on pedigree information are the same for all the members of the same family whereas those from model 2 based on SNP vary within families (Figure 3B). Part of the variation within families could be captured by SNP information, resulting in consistent improvement on the estimation of phenotypes (Table 7). Similar results were observed for other traits.

Because most elements of the realized relationship matrix based on SNP data are non-zero, sparse matrix techniques [25,33] could not be used neither to invert the **G **matrix nor to solve the mixed model equation. This resulted in much longer computing time to estimate variance components based on the realized relationship matrix. Therefore, we had to use the computationally tractable approach that was modified from the original approach. However, the estimated variance components for family, cage and polygenic effects were mostly consistent across the MCMC process. Therefore, we did not expect very different results when using the modified version.

In model 3, covariance between SNP was negligible probably because the model had a better fit when less dependent SNP were selected. However, this was not the case with model 4 because additive and dominance effects for a SNP were always fitted together whether they were correlated or not. This would cause a negative covariance between SNP effects, and overestimation of total phenotypic variance. When covariance between SNP is explicitly modelled, better estimates can be obtained although there is a risk of overparameterization in model 4.

## Conclusions

In conclusion, the proposed method implementing a realized relationship matrix based on aggregate SNP information is useful to genetically dissect complex traits especially when there are confounding factors between genetic and non-genetic effects. Resulting variance components are less biased and more accurate. A further analysis could be carried out using the proposed Bayesian approach to disentangle additive genetic and dominance effects. This novel strategy may help to understand the architecture of various complex traits.

## Competing interests

The authors declare that they have no competing interests.

## Authors' contributions

All authors conceived the idea, contributed to the study design, and method developments. SHL undertook the analysis. All authors drafted the manuscript and approved the final manuscript.

## Appendix A

### Relatedness estimation

Following Oliehoek et al. [18], the probability of genotypes for individual *i *and *j *being identical by state at locus *l *is,

(A1)

where *f*_*ij *_is the relatedness coefficients between *i *and *j*, and *H*_*l *_is the homozygosity at the locus *l *in the base population.

When giving  can be derived from (A1). Therefore,

(A2)

When multiple marker loci are used,

(A3)

Due to dependency of the estimates on the allele frequency at each locus, a different weight should be given to each locus. Following Oliehoek et al. [18], the weighted relatedness estimates are,

where  and *W *is the sum of the weights over all *L *loci.

The term *H*_*l *_is not usually known, so has to be estimated. Oliehoek et al. [18] introduced an equal drift estimator for obtaining *H*_*l *_assuming the increase in coancestry since the base population is equal at all loci.

(A4)

where *hz*_*l *_is expected homozygosity at locus l, and *hz*_*min *_is expected homozygosity at the locus having the lowest homozygosity. Expected homozygosity is calculated from estimated allele frequencies.

## Appendix B

### Empirical Bayesian approach

Concerning model 3 and 4, the number of significant SNPs (*n*_*q*_) and an indicator vector of their positions (**ρ**) are unknown parameters to be estimated. Reversible jump Markov chain Monte Carlo (RJMCMC) is used to obtain the posterior distribution of *n*_*q *_and **ρ**. During the MCMC procedure, maximum likelihood estimates are obtained for **β, f, c, g**, α, δ, , ,  and  (**Θ **) given sampled *n*_*q *_and **ρ **in every round. This is an empirical Bayesian approach [34,35]. The posterior probability of the parameters can be written as,

where *pr*(**y**|*n*_*q*_, ρ, **Θ **) is the likelihood of the observed phenotypes given the sampled variables, *pr*(*n*_*q*_, **ρ**, **Θ **) is the joint prior probability of the variables, and the denominator is summed over the probabilities of all possible parameter states. If the parameter states are many, a MCMC method can be an efficient tool to obtain the posterior distribution for the parameters as used in this study. When varying the number of SNP in the model, the model dimension varies. A Metropolis-Hastings sampler cannot properly infer the correct distribution unless the model dimension is fixed. However, a RJMCMC [36] can deal with all possible states across different model dimensions according to the proper acceptance ratio, and give the correct posterior distribution [37].

In this study, SNP-in and SNP-out step are used for moving the Markov chain across different model dimensions. With a proposal probability, a SNP can be newly added to the model (in) or excluded from the model (out). When adding a SNP (*n *_*q *_+ 1), its position is uniformly sampled from all unoccupied putative positions across the region, and included in the model. When deleting a SNP, one of the occupied SNP in the model is uniformly sampled, and excluded from the model. The parameters for the new model are estimated using ML. The proposal of adding or deleting a SNP is accepted with an acceptance ratio,

The first term in the right hand side is the posterior density consisting of the likelihood and the prior, and the second and third term are the proposal probability of adding or deleting a SNP from the model. *pr*(*n*_*q*_*|*n*_*q*_) is a proposal probability of changing the number of SNP in the model from *n*_*q *_to *n*_*q*_*, and *J *is the Jacobian of the transformation function probability from the current model to the other. Because adding or deleting a SNP in the method is the identity transformation, *J *is 1 [28,38,39]. The prior of the number of SNP, e.g. *pr*(*n*_*q*_), has a Poisson distribution with mean *μ*_*n *_=1. This assumes that there is no prior information available for the number of SNP, and this is a conservative way of detecting SNP associated with QTL, avoiding false positives. A flat uniform prior is used for **ρ**.

In order to remedy the computational burden due to dense **G, **the process is slightly modified. In the process, the log-likelihood of the observed phenotypes is conditional on the variance covariance matrix (*V*) with , ,  and  that are already estimated in model 2. The log-likelihood can be obtained as,

In every 1000 rounds, REML estimates for **α **and **δ **are obtained given *n*_*q *_and **ρ**, which assumes normal prior. It is noted that in this case, **α **and **δ **are random effects and have variances  and .

## References

[B1] Meuwissen THE Hayes BJ Goddard ME Prediction of total genetic value using genome-wide dense marker maps Genetics 2001 157 1819 1829 11290733 10.1093/genetics/157.4.1819 PMC1461589

[B2] Wray NR Goddard ME Visscher PM Prediction of individual genetic risk to disease from genome-wide association studies Genome Res 2007 17 1520 1528 10.1101/gr.6665407 17785532 PMC1987352

[B3] Risch N Merikangas K The future of genetic studies of complex human diseases Science 1996 273 1516 1517 10.1126/science.273.5281.1516 8801636

[B4] Gillian T Genotype versus phenotype: Human pigmentation Forensic Science International: Genetics 2007 1 105 110 10.1016/j.fsigen.2007.01.005 19083738

[B5] Lander ES Schork NJ Genetic dissection of complex traits Science 1994 265 2037 2048 10.1126/science.8091226 8091226

[B6] Andersson L Georges M Domestic-animals genomics: Deciphering the genetics of complex traits Nat Rev Genet 2004 5 202 212 10.1038/nrg1294 14970822

[B7] Henderson CR Applications of linear models in animal breeding 1984 University of Guelph, Guelph

[B8] Henderson CR Best linear unbiased estimation and prediction under a selection model Biometrics 1975 31 423 447 10.2307/2529430 1174616

[B9] Patterson HD Thompson R Recovery of interblock information when block sizes are unequal Biometrika 1971 58 545 554 10.1093/biomet/58.3.545

[B10] Lange K Westlake J Spence MA Extensions to pedigree analysis. III. Variance components by the scoring method Ann Hum Genet 1976 39 485 491 10.1111/j.1469-1809.1976.tb00156.x 952492

[B11] Sellers TA Weaver TW Phillips BP Altmann M Rich SS Environmental factors can confound identification of a major gene effect: Results from a segregation analysis of a simulated population of lung cancer families Genet Epidemiol 1998 15 251 262 10.1002/(SICI)1098-2272(1998)15:3<251::AID-GEPI4>3.0.CO;2-7 9593112

[B12] Goddard ME Hayes BJ Mapping genes for complex traits in domestic animals and their use in breeding programmes Nat Rev Genet 2009 10 381 391 10.1038/nrg2575 19448663

[B13] McCarthy MI Abecasis GR Cardon LR Goldstein DB Little J Ioannidis JPA Hirschhorn JN Genome-wide association studies for complex traits: consensus, uncertainty and challenges Nat Rev Genet 2008 9 356 369 10.1038/nrg2344 18398418

[B14] Visscher PM Sizing up human height variation Nat Genet 2008 40 489 490 10.1038/ng0508-489 18443579

[B15] Lee SH van der Werf JHJ Hayes BJ Goddard ME Visscher PM Predicting unobserved phenotypes for complex traits from whole-genome SNP data PLoS Genet 2008 4 e1000231 10.1371/journal.pgen.1000231 18949033 PMC2565502

[B16] Visscher PM Medland SE Ferreira MAR Morley KI Zhu G Cornes BK Montgomery GW Martin NG Assumption-free estimation of heritability from genome-wide identity-by-descent sharing between full siblings PLoS Genet 2006 2 e41 10.1371/journal.pgen.0020041 16565746 PMC1413498

[B17] Lynch M Ritland K Estimation of pairwise relatedness with molecular markers Genetics 1999 152 1753 1766 10430599 10.1093/genetics/152.4.1753 PMC1460714

[B18] Oliehoek PA Windig JJ van Arendonk JAM Bijma P Estimating relatedness between individuals in general populations with a focus on their use in conservation programs Genetics 2006 173 483 496 10.1534/genetics.105.049940 16510792 PMC1461426

[B19] Visscher PM Macgregor S Benyamin B Zhu G Gordon S Medland S Hill WG Hottenga J-J Willemsen G Boomsma DI Liu Y-Z Deng H-W Montgomery GW Martin NG Genome partitioning of genetic variation for height from 11,214 sibling pairs Am J Hum Genet 2007 81 1104 1110 10.1086/522934 17924350 PMC2265649

[B20] Hayes BJ Visscher PM Goddard ME Increased accuracy of artificial selection by using the realized relationship matrix Genet Res 2009 91 47 60 10.1017/S0016672308009981 19220931

[B21] Valdar W Solberg LC Gauguier D Burnett S Klenerman P Cookson WO Taylor MS Rawlins JNP Mott R Flint J Genome-wide genetic association of complex traits in heterogeneous stock mice Nat Genet 2006 38 879 887 10.1038/ng1840 16832355

[B22] Valdar W Solberg LC Gauguier D Cookson WO Rawlins JNP Mott R Flint J Genetic and environmental effects on complex traits in mice Genetics 2006 174 959 984 10.1534/genetics.106.060004 16888333 PMC1602068

[B23] Falconer DS Mackay TFC Introduction to quantitative genetics 1996 4 Longman 10.1093/genetics/167.4.1529 PMC1471025 15342495

[B24] Gilmour AR Cullis BR Welham SJ Thompson R ASREML reference manual 2004 New South Wales, Australia: Orange Agriculture Institute

[B25] Gilmour AR Thompson R Cullis BR Average information REML: an efficient algorithm for variance parameter estimation in linear mixed models Biometrics 1995 51 1440 1450 10.2307/2533274

[B26] Purcell S Neale B Todd-Brown K Thomas L Ferreira MAR Bender D Maller J Sklar P de Bakker PIW Daly MJ Sham PC PLINK: a tool set for whole-genome association and population-based linkage analyses Am J Hum Genet 2007 81 559 575 10.1086/519795 17701901 PMC1950838

[B27] Sillanpää MJ Gasbarra D Arjas E Comment on "On the Metropolis-Hastings acceptance probability to add or drop a quantitative trait locus in Markov chain Monte Carlo-based Bayesian analyses" Genetics 2004 167 1037 10.1534/genetics.103.025320 15238553 PMC1470884

[B28] Jannink J-L Fernando RL On the Metropolis-Hastings acceptance probability to add or drop a quantitative trait locus in Markov chain Monte Carlo-based Bayesian analyses Genetics 2004 166 641 643 10.1534/genetics.166.1.641 15020452 PMC1470712

[B29] Meuwissen TH Hayes BJ Goddard ME Prediction of total genetic value using genome-wide dense marker maps Genetics 2001 157 1819 1829 11290733 10.1093/genetics/157.4.1819 PMC1461589

[B30] O'Hara RB Sillanpaa MJ A review of Bayesian variable selection methods: What, how and which Bayesian Analysis 2009 4 85 118 10.1214/09-BA403

[B31] Meuwissen T Solberg T Shepherd R Woolliams J A fast algorithm for BayesB type of prediction of genome-wide estimates of genetic value Genet Sel Evol 2009 41 2 10.1186/1297-9686-41-2 19284681 PMC2637029

[B32] Spiegelhalter DJ BUGS 0.5 Bayesian inference using Gibbs sampling manual (version II) 1996 Cambridge: MRC, Biostatistics Unit

[B33] Duff IS Erisman AM Reid JK Direct method for sparse matrix 1989 Oxford, Clarendon Press

[B34] Casella G Empirical Bayes Gibbs sampling Biostatistics 2001 2 485 500 10.1093/biostatistics/2.4.485 12933638

[B35] Lee SH Van der Werf JHJ Simultaneous fine mapping of multiple closely linked quantitative trait loci using combined linkage disequilibrium and linkage with a general pedigree Genetics 2006 173 2329 2337 10.1534/genetics.106.057653 16751664 PMC1569695

[B36] Green P Reversible jump Markov chain Monte Carlo computation and Bayesian model determination Biometrika 1995 82 711 732 10.1093/biomet/82.4.711

[B37] Sorensen D Gianola D Likelihood, Bayesian, and MCMC methods in quantitative genetics 2002 New York: Springer

[B38] Yi N Xu S Bayesian mapping of quantitative trait loci under the identity-by-descent-based variance component model Genetics 2000 156 411 422 10978304 10.1093/genetics/156.1.411 PMC1461251

[B39] Sillanpää MJ Arjas E Bayesian mapping of multiple quantitative trait loci from incomplete inbred line cross data Genetics 1998 148 1373 1388 9539450 10.1093/genetics/148.3.1373 PMC1460044

